# Behavioral and Pharmacokinetic Profile of Indole-Derived Synthetic Cannabinoids JWH-073 and JWH-210 as Compared to the Phytocannabinoid Δ^9^-THC in Rats

**DOI:** 10.3389/fnins.2018.00703

**Published:** 2018-10-23

**Authors:** Libor Uttl, Ewa Szczurowska, Kateřina Hájková, Rachel R. Horsley, Kristýna Štefková, Tomáš Hložek, Klára Šíchová, Marie Balíková, Martin Kuchař, Vincenzo Micale, Tomáš Páleníček

**Affiliations:** ^1^Department of Experimental Neurobiology, National Institute of Mental Health, Klecany, Czechia; ^2^Department of Physiology, Faculty of Science, Charles University, Prague, Czechia; ^3^Forensic Laboratory of Biologically Active Compounds, Department of Chemistry of Natural Compounds, University of Chemistry and Technology Prague, Prague, Czechia; ^4^Institute of Forensic Medicine and Toxicology, First Faculty of Medicine, Charles University and General University Hospital, Prague, Czechia; ^5^Department of Biomedical and Biotechnological Sciences, Section of Pharmacology, University of Catania, Catania, Italy; ^6^Third Faculty of Medicine, Psychiatric Clinic, Charles University, Prague, Czechia

**Keywords:** synthetic cannabinoids, Δ^9^-THC, pharmacokinetics, behavior, JWH-073, JWH-210

## Abstract

Synthetic cannabinoid compounds are marketed as “legal” marijuana substitutes, even though little is known about their behavioral effects in relation to their pharmacokinetic profiles. Therefore, in the present study we assessed the behavioral effects of systemic treatment with the two synthetic cannabinoids JWH-073 and JWH-210 and the phytocannabinoid Δ^9^-THC on locomotor activity, anxiety-like phenotype (in the open field) and sensorimotor gating (measured as prepulse inhibition of the acoustic startle response, PPI), in relation to cannabinoid serum levels. Wistar rats were injected subcutaneously (sc.) with JWH-073 (0.1, 0.5, or 5 mg/kg), JWH-210 (0.1, 0.5, or 5 mg/kg), Δ^9^-THC (1 or 3 mg/kg) or vehicle (oleum helanti) in a volume of 0.5 ml/kg and tested in the open field and PPI. Although JWH-073, JWH-210, Δ^9^-THC (and its metabolites) were confirmed in serum, effects on sensorimotor gating were absent, and locomotor activity was only partially affected. Δ^9^-THC (3 mg/kg) elicited an anxiolytic-like effect as suggested by the increased time spent in the center of the open field (*p* < 0.05). Our results further support the potential anxiolytic-like effect of pharmacological modulation of the endocannabinoid system.

## Introduction

Synthetic cannabinoids (SCs) are substances referred to as cannabinoid CB1 and/or CB2 receptor ligands that were originally developed as research tools to assess the endocannabinoid system (ECS) pharmacology and to examine the cannabinoid CB1 and CB2 receptors (Wiley et al., 2012). Since the beginning of 2000s, they appeared on the drug market worldwide under exotic brand names such as “Spice,” “Jamaican Spirits,” or “K2” and have become popular for their psychoactive and euphoric cannabis-like effects and also for their ability to escape detection by standard cannabinoid screening tests (Fattore and Fratta, 2011).

Most synthetic cannabinoids are highly lipophilic compounds which easily cross the blood brain barrier, and they typically exhibit higher affinities (in some cases 100 times higher) for central and peripheral cannabinoid CB1 receptors than the psychoactive phytocannabinoid Δ9-tetrahydrocannabinol (Δ^9^-THC; Ki = 41 ± 2 nM) (Huffman et al., 2005). Therefore, they could induce stronger cannabimimetic effects such as anti-nociception, catalepsy, hypothermia, cognitive impairment, altered sensory perception and psychotic reactions (Huffman et al., 2005; Kucerova et al., 2014; Fattore, 2016; Tait et al., 2016).

Unlike *cannabis*, which has a reputation as fairly benign substance, the SCs have been associated with systemic toxicities including myocardial infarction (Schwartz et al., 2015), ischemic strokes (Freeman et al., 2013; Takematsu et al., 2014), seizures (Schneir and Baumbacher, 2012; Schwartz et al., 2015), acute kidney injury (Buser et al., 2014) and sudden death; thus their abuse has become a substantial social and public health issue (Behonick et al., 2014; Castaneto et al., 2014, 2015; Tai and Fantegrossi, 2014).

One of the most frequently occurring SCs identified in specimens from users belongs to the group of indole-derivatives or aminoalkylindoles family (the “JWH” series) (Uchiyama et al., 2011; Carroll et al., 2012). Among these, JWH-073 (1-butyl-1H-indol-3-yl)-1-naphthalenyl-methanone) has four-fold higher binding affinity toward central CB1 receptors (Ki that ranging from 8.9 ± 1.8 to 12.9 ± 3.4 nM) than Δ^9^-THC (Wiley et al., 1998; Aung et al., 2000; Brents et al., 2012) and is biotransformed *in vivo* into monohydroxylated metabolites that retain significant affinity and activity at cannabinoid CB1 receptors (Brents et al., 2012). *In vivo* animal studies report that JWH-073 reproduces the typical “tetrad” effects of Δ^9^-THC such as hypothermia, analgesia, hypolocomotion, akinesia (Wiley et al., 1998; Brents et al., 2012; Marshell et al., 2014), as well as impaired sensorimotor responses, seizures and aggressiveness (Ossato et al., 2016). In human studies agitation, hallucinations, confusions and alterations in cognitive abilities have been reported (Papanti et al., 2013; Zawilska and Wojcieszak, 2014).

JWH-210 is a newer compound detected in the “marijuana alternatives”, which has a high binding affinity toward central cannabinoid CB1 receptors (Ki = 0.46 ± 0.03 nM). In comparison to other cannabinoids, it has 20 times higher affinity to CB1 than JWH-073 and 100 times higher than Δ^9^-THC; thus it reproduces a stronger “tetrad” effects in rodents as well as nausea, seizures and cardiovascular impairment in humans (Dogan et al., 2016; Hermanns-Clausen et al., 2016; Tait et al., 2016).

Given that these drugs have been found in severe poisonings in humans, including fatalities, we assessed the behavioral and pharmacokinetic profile of JWH-073 and JWH-210 as compared to Δ^9^-THC in rats. More specifically, their potential anxiogenic- and/or anxiolytic-like effects were investigated in the open field test (OFT), an unconditioned test based on spontaneous behavior of animals which is usually used to assess anxiety, as well as exploration and locomotor activity (Micale et al., 2013b). Given that chronic *cannabis* use in healthy individuals or systemic treatment with CB1/CB2 agonists (i.e., Δ^9^-THC or WIN55,212-2) in laboratory animals may affect sensorimotor gating (Kucerova et al., 2014), the prepulse inhibition (PPI) of the acoustic startle response (ASR) was also assessed (Micale et al., 2013a; Horsley et al., 2018). Alongside this, the pharmacokinetic profiles of JWH-073, JWH-210, Δ^9^-THC (as well as Δ^9^-THC metabolites 11-OH-THC and THC-COOH) in serum were also evaluated.

## Materials and methods

### Animals

All experiments were carried out on male Wistar rats (200–250 g) (VELAZ, Czech Republic). Animals were housed in pairs in a 12 h light/dark cycle regime at 22 ± 2°C and water and standard diet *ad libitum*. Before the behavioral testing, animals (*n* = 10 per group) were acclimatized for 7–10 days during which they were handled four times and weighed twice. Experiments and measurements were conducted during the light phase of the cycle (between 8:00 and 14:00 h). In order to minimize the total number of animals used across experiments, rats from behavioral experiments were subsequently used for pharmacokinetic analyses (*n* = 8 per one time point). All experiments respected the Guidelines of the European Union (86/609/EU) and the National Committee for the Care and Use of Laboratory Animals (Czech Republic), and were according to Guidelines of the European Union (86/609/EU). The protocol was approved by the National Committee for the Care and Use of Laboratory Animals (Czech Republic) under the number: MEYSCR-27527/2012-31.

### Drugs and chemicals

The SCs JWH-073 (1-butylindol-3-yl)-naphthalen-1-ylmethanone and JWH-210 (4-ethyl-1-naphtalenyl) (1-phenyl-1H-indol-3-yl)-methanone were purchased *via* the internet and subsequently purified by Alfarma s.r.o (Czech Republic). The resulting compounds were analyzed for purity, JWH-073 99.48% and JWH-210 97.84% (analyzed by infrared spectroscopy), and in the form of a free base were dissolved in pharmaceutical grade sunflower oil (oleum helanti) and administered subcutaneously (sc.) at the doses of 0.1, 0.5, or 5.0 mg/kg in a volume of 0.5 ml/kg. The phytocannabinoid Δ^9^-THC 99.3% (THC-Pharm GmbH) was dissolved in oleum helanti and administered sc. at the dose of 1 or 3 mg/kg in a volume of 0.5 ml/kg. Control animals were treated with the corresponding amounts of sunflower oil as vehicle. The doses of the SCs were selected according to the reports from users on the internet and according to the potency of similar compounds that have been tested in preclinical experiments (Cha et al., 2015; Gatch and Forster, 2016; Ossato et al., 2016). The doses of Δ^9^-THC were selected based on our previous results focusing on its behavioral and pharmacokinetic effects induced by different routes of administration (Micale et al., 2013a; Hlozek et al., 2017).

### Pharmacokinetics

#### Determination of JWH-073 and JWH-210 levels in serum samples

Different groups of rats (*n* = 8 per group) were treated sc. with JWH-073 (0.5 mg/kg), JWH-210 (0.5 mg/kg) and subsequently decapitated after 30 min, 1, 2, 4, 8, or 24 h. Serum samples were collected and stored at −20°C. These samples were analyzed after extensive optimization and validation of the sample preparation procedure according to the 2001 FDA Guidance using LC-MS method. Serum sample preparation consists of a protein precipitation and was following: (1) 800 μL 0.1% solution of formic acid in acetonitrile (v/v) was cooled down for 30 min at −20°C; (2) 200 μL of serum was added to the cooled solution and immediately mixed in a Bullet Blender Storm homogenizer (Next Advance, United States) for 5 min (speed 4); (3) centrifugation for 10 min (14,000 RPM) at 5°C; (4) evaporation of 800 μL supernatant to dryness (Centrivap Concentrator); and (5) reconstitution with 0.1% formic acid in water/acetonitrile, 80/20 (v/v). Prior to analysis by LC-MS, all samples were vortexed and centrifuged. LC-MS analysis: the samples in this section were analyzed using UHPLC-MS/MS instrumentation (1,290 Infinity Agilent Technologies Agilent 6460 Triple Quadrupole LC/MS with Agilent Jet Stream electrospray ionization source). A column Agilent Zorbax Eclipse RRHD (50 × 2.1 mm, 1.8 μm) with a pre-column was used for a chromatographic separation with gradient elution in system of 0.1% (v/v) formic acid (mobile phase A) and acetonitrile (mobile phase B). Data were acquired in positive electrospray ionization (ESI) mode by a multiple reaction monitoring method (MRM). JWH-073 and JWH-210 were quantified using an external matrix-matched calibration (US FDA. Guidance for Industry: Bioanalytical Method Validation. US FDA, Center for Drug Evaluation and Research, MD, USA 2001). Limit of detection (LOD) and quantification (LOQ) were for both drugs 0.05 ng/ml and 1 ng/ml, respectively.

#### Determination of Δ^9^-THC levels in serum samples

Different groups of rats (*n* = 6 per group) were treated sc. with Δ^9^-THC (3 mg/kg) and subsequently decapitated after 30 min, 1, 2, 4, 8, or 24 h. Serum were collected and stored at −20°C. Δ^9^-THC were determined by an in-house validated and certified GC-MS method (certified by Police Presidium of the CR, ref. no.: PPR-31123-7/CJ-2015-990530/ evidence no.: 16/2015). A total of 10 μl of deuterated THC-d3/11-OH-THC-d3/THC-COOH-d3 (5 ng/μl) internal standard solution was added to each 1.0 ml sample of serum. Serum was diluted with a 4 ml sodium acetate buffer with a pH of 4.0 (0.01 mol/l). Serum phytocannabinoid Δ^9^-THC was extracted with SPE columns (Bond-ELUT, 130 mg, Agilent Technologies), eluted with hexal/ethyl acetate (1:4 v/v) and dried under a nitrogen gas stream in a 400 μl glass insert placed in a 1.5 glass vial. The samples were derivatized with 100 μl of N-Methyl-N-(trimethylsilyl) trifluoroacetamide (MSTFA) for 20 min at 80°C. Quantification of extracted Δ^9^-THC was performed by gas chromatography-mass spectrometry (GC-MS) (GC7860/5742CMSD, Agilent Technologies) using electron impact ionization in the selective ion mode (THC: m/z 386; THC-d3: m/z 389; 11-OH-THC: m/z 371; 11-OH-THC-d3: m/z 374; THC-COOH: m/z 371; THC-COOH-d3: m/z 374). Calibration curve ranges were prepared by spiking drug-free bovine serum at concentrations (1) 2–200 ng/ml THC, 11-OH-THC, and THC-COOH; (2) 100–1,000 ng/ml THC, 11-OH-THC and THC-COOH. Limit of detection (LOD) and quantification (LOQ) were 1 and 2 ng/ml, respectively. The spikes were vortexed and treated identically to the experimental samples (Hlozek et al., 2017).

### Behavioral experiments

All behavioral experiments were performed 1 h after sc. drugs administration.

#### Open field

A square black plastic open field arena (68 × 68 × 30 cm) was placed in a soundproof and diffusely lit room. Each of animals was placed into the center of the arena, in a novel unfamiliar environment, and 1 h after drug administration the behavior was video-recorded for 30 min using the system EthoVision Color pro v. 3.0 (Noldus, NL). Locomotor activity was subsequently analyzed within 5 min blocks/time intervals (1–6). The calculation of the data was performed in the EthoVision software and corrected (smoothed) for movement deviations of < 3 cm. Initially total distance traveled per time block was calculated and data were plotted in the graphs. To evaluate the spatial characteristics of the locomotor activity such as thigmotaxis and time spent in the center of arena, the arena was virtually divided into 5 × 5 identical square zones with 16 located peripherally and nine centrally. Frequency (*f*) of appearances of the animal in different zones of the arena was used to calculate thigmotaxis (*i*) (*i* = ∑*f*_*peripheralzones*_ */* ∑*f*_*allzones*_) which is a number (value varying from 0 to 1) indicating the probability of appearance in peripheral zones. Time spent in the center of the arena (T_center_) was calculated T_center_ = ∑*time*_centralzones_ (Balikova et al., 2014; Horsley et al., 2016; Palenicek et al., 2016; Tyls et al., 2016; Hlozek et al., 2017; Sichova et al., 2017; Stefkova et al., 2017).

#### Prepulse inhibition (PPI) of acoustic startle response (ASR)

The PPI of ASR took place in two ventilated startle chambers (SR-LAB, San Diego Instruments, California, USA) which were calibrated to ensure equivalent stabilimeter sensitivity between the chambers. The test consists of acclimatization and two sessions, as previuosly described (Direnberger et al., 2012; Palenicek et al., 2016; Tyls et al., 2016; Hlozek et al., 2017; Sichova et al., 2017; Stefkova et al., 2017). Briefly, acclimatization was performed 2 days before the test, when drug-free rats were habituated in 5 min session with five presentations of pulse alone stimuli (115 dB/20 ms) over background white noise (75 dB). On the day of test, the compounds or vehicle were administered sc. 1 h prior to PPI/ASR testing. After acclimatization (5 min with 75 dB background noise), the test started with a short session of six 40 ms 125 dB pulse trials to establish baseline ASR. It was followed by the second session consisting of trials presented in a pseudorandom order: (1) single pulse alone: 40 ms 125 dB; (2) trial of prepulse-pulse: 20 ms prepulse of 83 dB presented 30, 60, and 120 ms (average 70 ms) before 40 ms 125 dB pulse; (C) 60 ms no stimulus. Finally, six 40 s 125 dB pulse trials were delivered. Habituation was calculated by the percentage reduction in ASR from the initial six, to the final six pulse trials. PPI was calculated as: [100–(mean prepulse–pulse trials/mean pulse alone trials)^*^100]. ASR was derived from mean pulse alone trials. Animals with an AVG response lower than 10 were excluded from further analysis as non-responders.

### Statistical analysis

To evaluate the effect on the locomotion measured in 5 min intervals, two-way repeated measures ANOVA analysis (factor 1: drug; factor 2: time intervals) was used in software system IBM SPSS version 22. Significant main effects and interaction two-way repeated measures ANOVAs were followed with pairwise comparisons using independent *t*-test. For repeated measures ANOVAs, where Mauchly's test of sphericity was significant, Greenhouse-Geisser [Greenhouse-Geisser estimate of sphericity (ε) < 0.75 or Huynh-Feldt (ε) >0.75] correction are reported. Degrees of freedom were rounded to whole number for presentational purposes. For independent *t*-test, where Levene's test for equality of variances was significant, statistics corrected for unequal variances are given *p* < 0.05 (two tailed) was considered the minimal criterion for statistical significance. For multiple comparisons, *t*-test was used with Bonferroni correction. The total length of the trajectory over 30-min, thigmotaxis and time in the center, ASR, habituation and PPI were analyzed by one-way ANOVA analysis by using software STATISTICA version 9.0. Where appropriate, ANOVA analyses were followed by the Tukey *post-hoc* test. Statistical significance was set at *p* < 0.05 for all analyses.

## Results

### Pharmacokinetics

**Figure 1:**
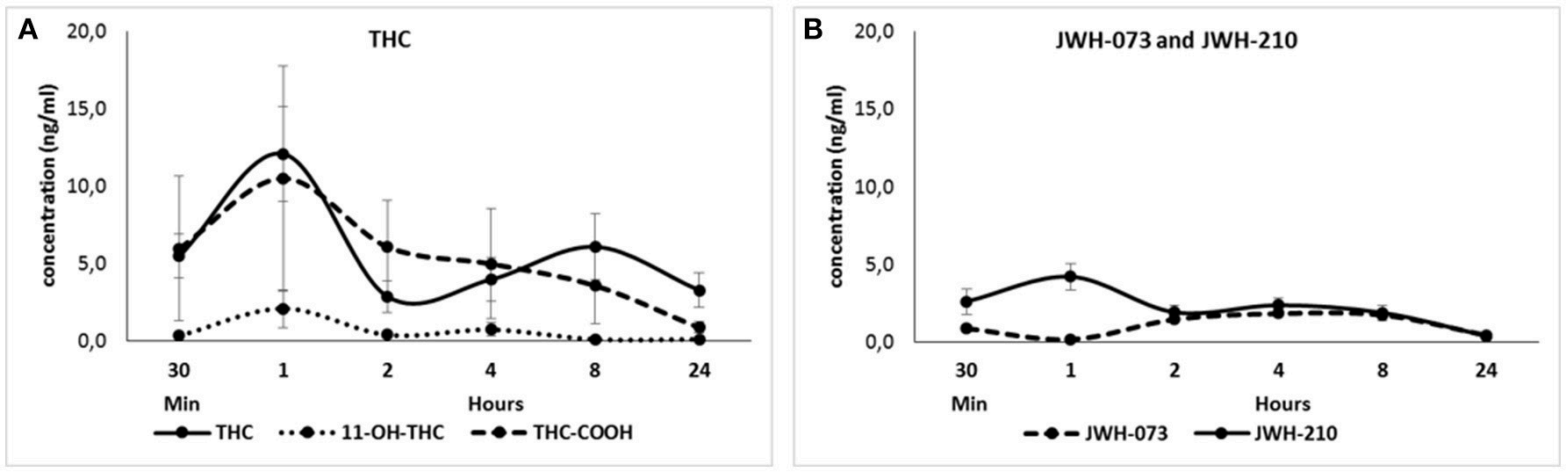
**(A)** Pharmacokinetic profile of Δ^9^-THC (3 mg/kg, sc.) and its metabolites 11-OH-THC and THC-COOH at different time point (30 min, 1, 2, 4, 8, or 24 h after treatment). Data are presented as mean ± SEM (*n* = 6 animals per group) of serum levels expressed in ng/ml. **(B)** Pharmacokinetic profile of JWH-073 (0.5 mg/kg, sc.) and JWH-210 (0.5 mg/kg, sc.) at different time point (30 min, 1, 2, 4, 8, or 24 h after treatment). Data are presented as mean ± SEM (*n* = 8 animals per group) of serum levels expressed in ng/ml.

Δ^9^-THC (3 mg/kg) and its metabolites 11-OH-THC and THC-COOH were detected within 24 h after sc. administration (Figure 1A). They reached the maximum serum concentration (Mean ± SEM; Δ^9^-THC: 12.1 ± 3.06 ng/ml; 11-OH-THC: 2.08 ± 1.21 ng/ml; THC-COOH: 10.5 ± 7.27 ng/ml) 1 h after the treatment. Second peak of Δ^9^-THC was observed after 8 h. JWH-073 (0.5 mg/kg) and JWH-210 (0.5 mg/kg) were detected within 24 h after sc. administration (Figure 1B). The maximum mean of JWH-073 serum concentration (1.84 ± 0.06 ng/ml) was attained 4 h after the treatment. JWH-210 reached the maximum serum concentration (4.20 ± 0.86 ng/ml) 1 h after administration and second peak of JWH-210 was detected after 4 h. 24 h after both cannabinoids JWH-073 (0.41 ± 0.19 ng/ml) and JWH-210 (0.38 ± 0.12 ng/ml) were slightly above the level of detection (LOD = 0.05 ng/ml; LOQ = 1 ng/ml).

### Behavior

#### Open field test: total locomotor activity

**Figure 2:**
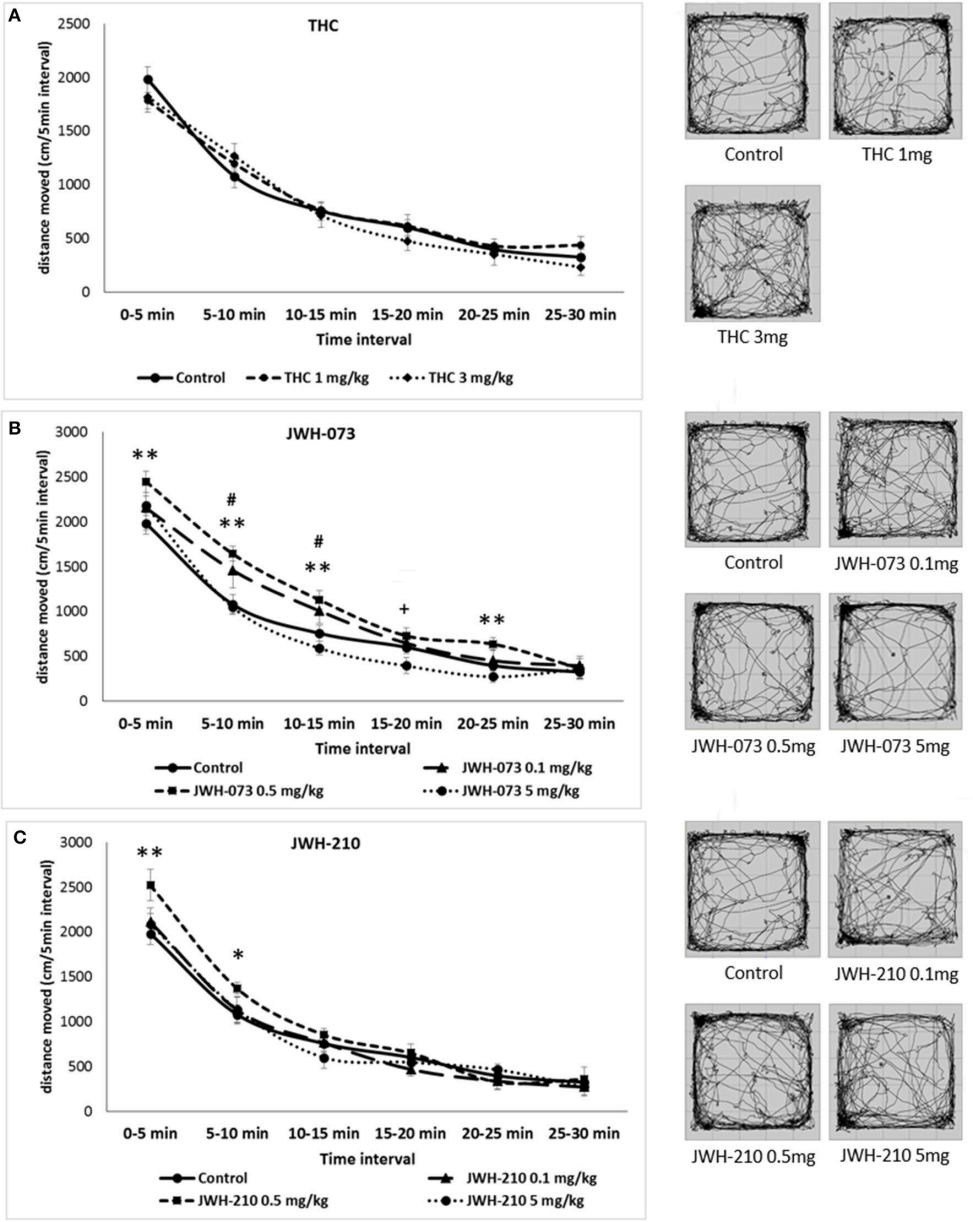
Open field test (OFT): Trajectory length (divided into 5-min blocks) and trajectory pattern over the entire 30 min period test. **(A)** Δ^9^-THC (1 or 3 mg/kg, sc.) error bars display ± 1 SEM. **(B)** JWH-073 (0.1, 0.5 or 5 mg/kg, sc.) error bars display ± 1 SEM. ^#^*p* < 0.05 for JWH-073 0.1 mg/kg, sc., ^**^p < 0.01 for JWH-073 0.5 mg/kg, sc. and ^+^*p* < 0.05 for JWH-073 5 mg/kg, sc. vs. vehicle group. **(C)** JWH-210 (0.1, 0.5, and 5 mg/kg, sc.), error bars display ± 1 SEM. JWH-210 (0.5 mg/kg, sc.) ^*^p < 0.05; JWH-210 (0.5 mg/kg, sc.) ^**^p < 0.01 vs. vehicle group.

Mauchly's test of sphericity was significant and Greenhouse-Geisser correction is presented for repeated measures, Mauchly's *W*_(14)_ = 0.44, *p* = 0.01. Analyses of locomotion in 5 min time intervals following Δ^9^-THC administration revealed a main effect of time interval [*F*_(4, 133)_ = 160.02, *p* < 0.001], but neither a main effect of drug nor drug × time interval interaction were found (Figure 2A). The locomotor activity of Δ^9^-THC and vehicle treated rats gradually decreased over the course of the test session, indicative of normal habituation.

For JWH-073 and JWH-210 Mauchly's tests of sphericity were significant [JWH-073: *W*_(14)_ = 0.35, *p* = 0.00; JWH-210: *W*_(14)_ = 0.50, *p* = 0.00] and Greenhouse-Geisser correction are presented for repeated measures JWH-073 and Huynh-Feldt correction are presented for repeated measures JWH-210. The SCs JWH-073 and JWH-210 had significant effect on time intervals [JWH-073: *F*_(3, 156)_ = 260.85, *p* < 0.001; JWH-210: *F*_(4, 204)_ = 261.91, *p* < 0.001]. Furthermore, there was drug effect for JWH-073 [*F*_(3, 46)_ = 3.76, *p* < 0.05], but not for JWH-210. The interaction between drug and time interval was significant for JWH-073 [*F*_(10, 156)_ = 1.88, *p* = 0.05], but not for JWH-210 [*F*_(11, 174)_ = 1.67, *p* = 0.08]. The locomotor activity gradually decreased in all treated groups suggesting that habituation was not attenuated in any of the treatments used (Figures 2B,C). At the dose of 0.1 mg/kg, JWH-073 significantly increased locomotor activity at the 5-10 and 10-15 min time blocks, minimum [*t*_(28)_ = 1.60, *p* < 0.05], but not in the others time blocks (with 0, 15, 20, and 25 min onset). Similarly, at the dose of 0.5 mg/kg it increased locomotion at 0–5, 5–10, 10–15, and 20–25 min onset, minimum [*t*_(28)_ = 2.44, *p* < 0.01]. By contrast, JWH-073 (5 mg/kg) reduced the locomotor activity at 15–20 min time block as compared to vehicle-treated animals [*t*_(28)_ = 1.74, *p* < 0.05; Figure 2B]. As described in Figure 2C, JWH-210 (0.5 mg/kg) treated rats showed a significant increased locomotor activity at 0 to 5 and 5 to 10 min time blocks as compared to vehicle-treated group, minimum [*t*_(28)_ = 1.79, *p* < 0.05].

**Figure 3:**
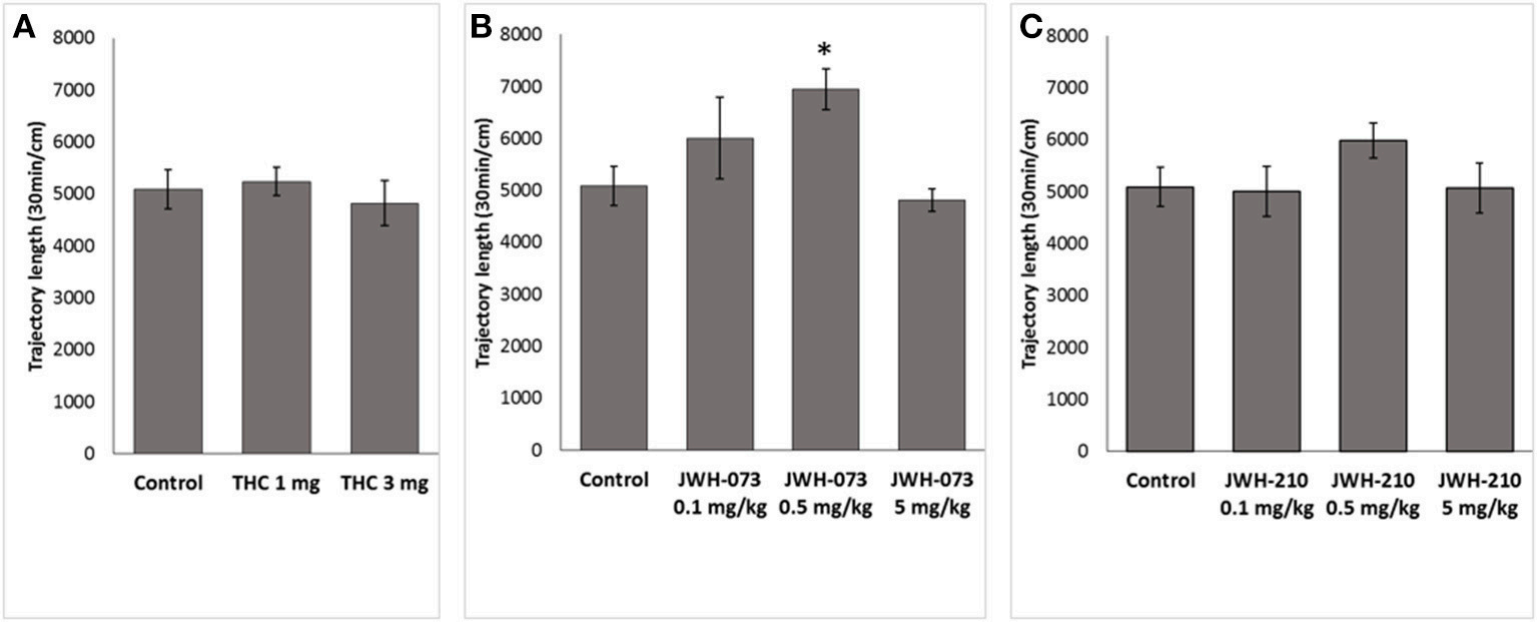
Total locomotor activity. **(A)** Δ^9^-THC (1 and 3 mg/kg, sc.), **(B)** JWH-073 (0.1, 0.5, and 5 mg/kg, sc.) and **(C)** JWH-210 (0.1, 0.5, and 5 mg/kg, sc.). Data are presented as mean ± SEM of distance traveled expressed in cm over the entire 30 min period test. ^*^p < 0.05 vs. vehicle group.

One-way ANOVA for total length of the trajectory over 30-min revealed a significant effect of JWH-073 treatment [*F*_(3, 46)_ = 3.7562, *p* < 0.05]. *Post-hoc* analysis showed that JWH-073 (0.5 mg/kg) significantly increased locomotor activity (*p* < 0.05) as compared to the control group (Figure 3B). Neither Δ^9^-THC [*F*_(2, 37)_ = 0.21685, *p* = 0.8, Figure 3A] nor JWH-210 [*F*_(3, 46)_ = 0.96950, *p* = 0.41, Figure 3C] affected the total locomotor activity.

#### Open field test: thigmotaxis and the time spent in the center of arena

**Figure 4:**
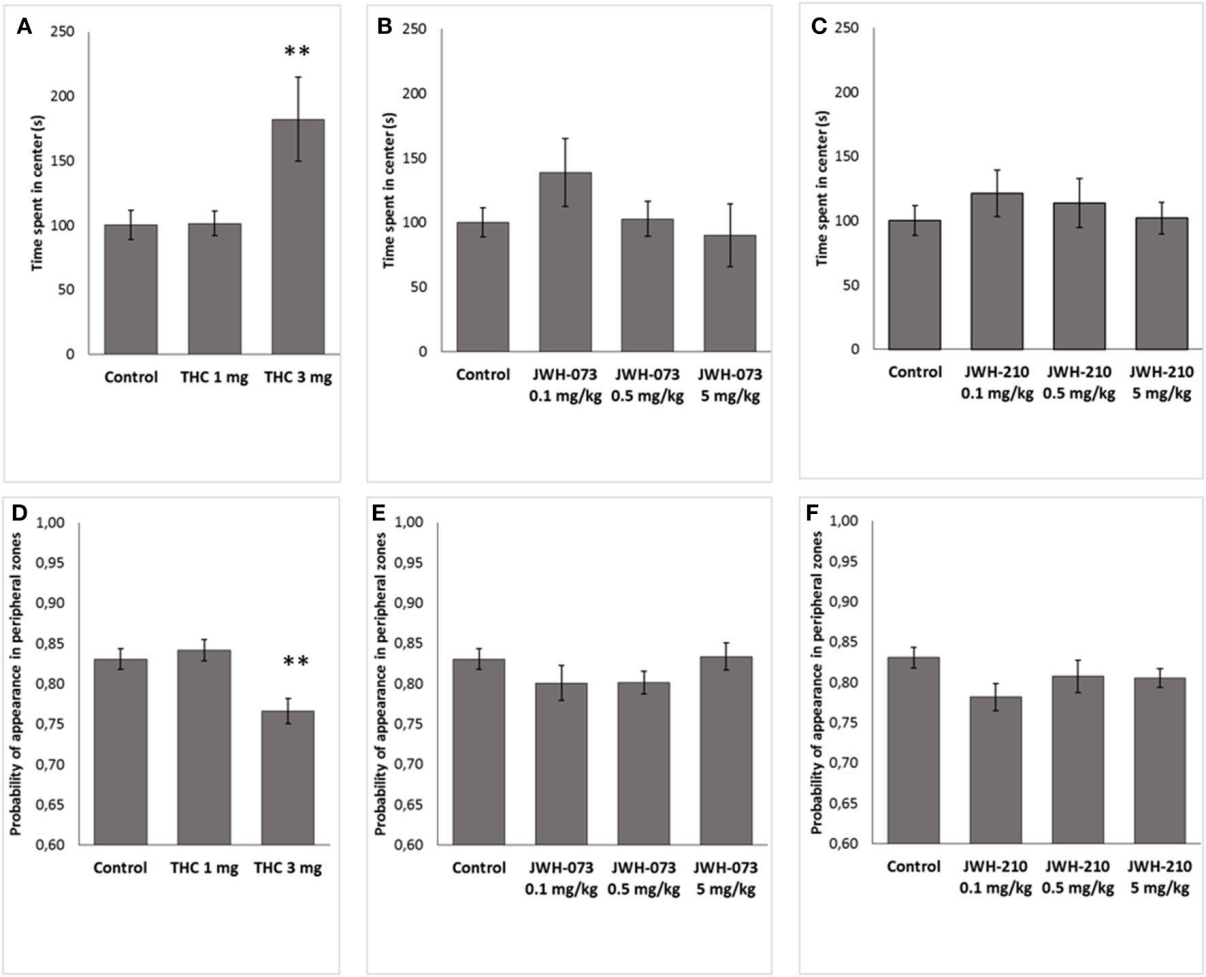
Time (s) spent in the center of open field arena. **(A)** Δ^9^-THC (1 and 3 mg/kg, sc.), **(B)** JWH-073 (0.1, 0.5, and 5 mg/kg, sc.) and **(C)** JWH-210 (0.1, 0.5, and 5 mg/kg, sc.). Probability of appearance in peripheral zones (thigmotaxis). **(D)** Δ^9^-THC (1 and 3 mg/kg, sc.), **(E)** JWH-073 (0.1, 0.5 and 5 mg/kg, sc.) and **(F)** JWH-210 (0.1, 0.5, and 5 mg/kg, sc.). Data are presented as mean ± SEM. ^**^p < 0.01 vs. vehicle treated group.

Δ^9^-THC significantly modified thigmotaxis [*F*_(2, 37)_ = 6.4791, *p* < 0.05] and the time in the central zones [*F*_(2, 37)_ = 6.0172, *p* < 0.001]. *Post-hoc* revealed that Δ^9^-THC (3 mg/kg) increased time spent in central zone (*p* < 0.01, Figure 4A) and decreased thigmotaxis (*p* < 0.01, Figure 4D), as compared to control animals. By contrast, neither JWH-073 nor JWH-210 modified the thigmotaxis [JWH-073 *F*_(3, 46)_ = 1.1572, *p* = 0.33, Figure 4E; JWH-210 *F*_(3, 46)_ = 1.8661, *p* = 0.14, Figure 4F] or the time spent in the central zones [JWH-073 *F*_(3, 46)_ = 1.1891, *p* = 0.32, Figure 4B; JWH-210 *F*_(3, 46)_ = 0.45117, *p* = 0.71, Figure 4C].

#### Prepulse inhibition (PPI) of acoustic startle response (ASR)

**Figure 5:**
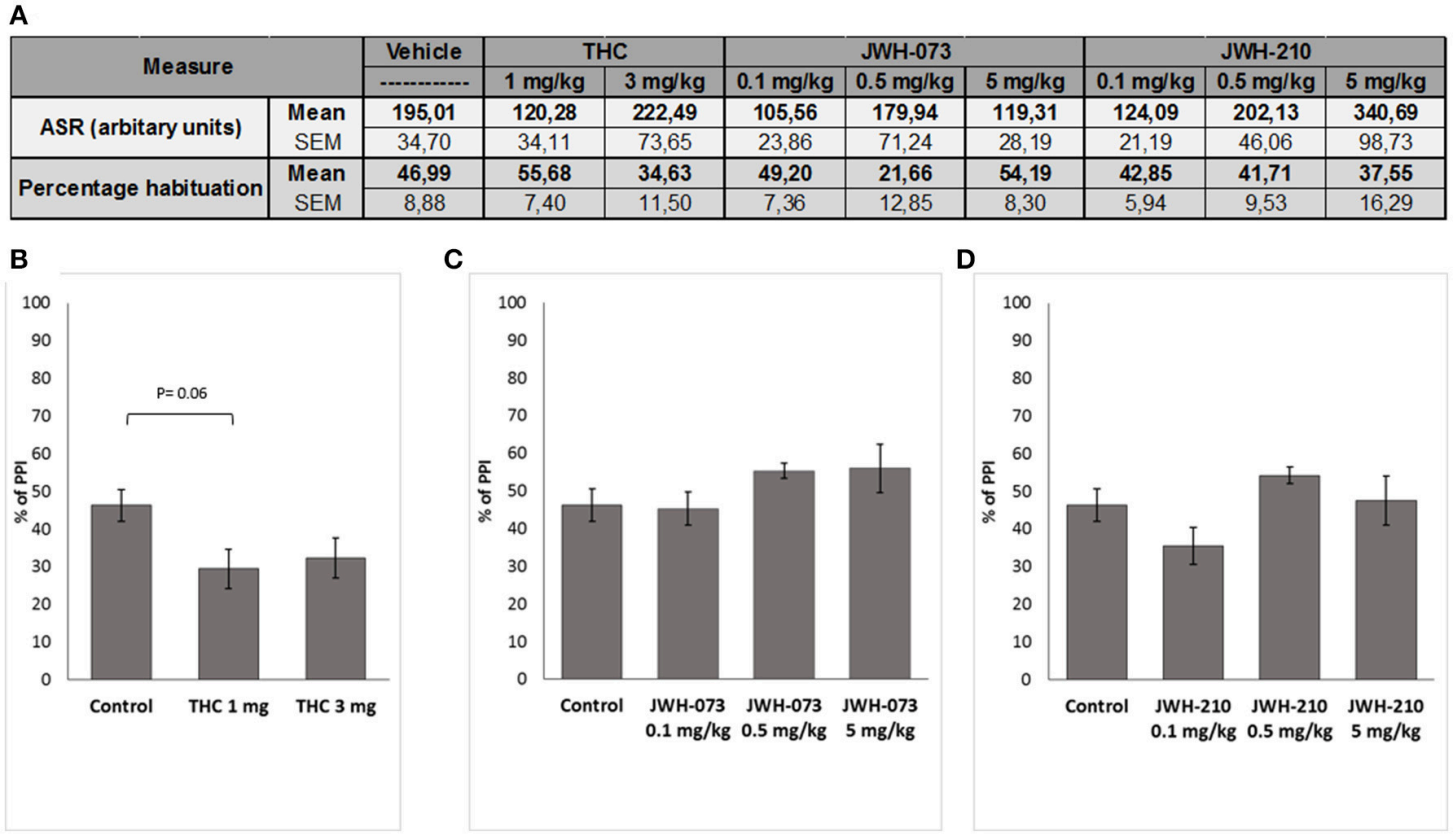
**(A)** Effect of Δ^9^-THC (1 or 3 mg/kg, sc.), JWH-073 (0.1, 0.5, and 5 mg/kg, sc.) and JWH-210 (0.1, 0.5 or 5 mg/kg, sc.) on acoustic startle response (ASR) and habituation. Effect of **(B)** Δ^9^-THC (1 or 3 mg/kg, sc.), **(C)** JWH-073 (0.1, 0.5, or 5 mg/kg, sc.) and **(D)** JWH-210 (0.1, 0.5, or 5 mg/kg, sc.) on prepulse inhibition. Data are presented as mean ± SEM regarding percentage (%) of prepulse inhibition.

ASR data were initially screened for non-responders (ASR < 10) leading to exclusion of following number of animals: JWH-073 (*n* = 4), JWH-210 (*n* = 5), Δ^9^-THC (*n* = 2) and controls (*n* = 3). Subsequent analyses revealed that none of the tested compounds affected the ASR (Figure 5A). Similarly, habituation data showed no significant treatment effect.

Although Δ^9^-THC treatment significantly affected PPI [*F*_(2, 32)_ = 3.6635, *p* < 0.05, Figure 5B], *post-hoc* analysis found a slight not significant decrease of PPI (*p* = 0.06) induced by Δ^9^-THC at the dose of 1 mg/kg. Neither JWH-073 [*F*_(3, 39)_ = 1.4218, *p* = 0.25, Figure 5C] nor JWH-210 [*F*_(3, 38)_ = 2.2994, *p* = 0.09, Figure 5D] affected the PPI.

## Discussion

In this study, we evaluated the behavioral effects and the pharmacokinetic profile of acute treatment with JWH-073, JWH-210 and Δ^9^-THC. The main findings of pharmacokinetic studies were as follows: JWH-073 had slow pharmacokinetics which peaked after 4 h, and was detectable at all measurement intervals with a temporary decrease 1 h after administration. JWH-210 had biphasic profile in serum, showing the highest peak and the second peak 1 and 4 h after administration, respectively. Furthermore, it was detectable at all intervals. The profile of Δ^9^-THC in serum was very similar to that of JWH-210: biphasic, with the highest peak at 1 h post-administration but the second peak later, at 8 h after administration. Metabolites from Δ^9^-THC (11-OH-THC and THC-COOH) had only one peak (1 h after the administration), and they were detectable as Δ^9^-THC at all intervals.

The main behavioral effect was that JWH-073 at the dose of 0.5 mg/kg increased the total locomotor activity in the OFT. Furthermore, rats treated with Δ^9^-THC (3mg/kg) spent more time in the center of OF arena, as index of anxiolytic-like effect. Sensorimotor gating, as measured by PPI, baseline startle (ASR) or habituation were not altered by the pharmacological treatment.

### Pharmacokinetics

The phytocannabinoid Δ^9^-THC (3 mg/kg) showed biphasic profile, in agreement with our previous results (Hlozek et al., 2017). A similar profile was also observed for JWH-210 and might be due to their partial release from subcutaneous tissue into the bloodstream, followed by their partial accumulation in adipose tissue; thus resulting in slow degradation over 24 h. By contrast, JWH-073 showed a major peak 4 h after the administration, suggesting its higher lipophilicity and slower release into the blood. Previous results have shown that Δ^9^-THC elicited different behavioral effects as well as different serum levels based on route of administration (Leighty, 1973; Deiana et al., 2012; Hlozek et al., 2017). In animal studies focusing on the effects of different routes of administration (i.e., intraperitoneal, intravenous or pulmonary) on SCs pharmacokinetic profile more rapid peaks and higher concentrations were detected (Marshell et al., 2014; Kevin et al., 2017; Malyshevskaya et al., 2017). In our study the detection of cannabinoid serum levels 24 h after the treatment is in agreement with previous studies showing their detection even for longer period (Schaefer et al., 2014; Hasegawa et al., 2015).

### Behavioral effects: open field and PPI

We found that acute treatment with JWH-073 at dose of 0.5 mg/kg, but not of 0.1 or 5 mg/kg significantly increased total trajectory in the open field test. However, treatment with JWH-210 (0.1–5 mg/kg) did not result in locomotor activity change. Our results are not consistent with those previously described showing that treatment with JWH-210 (0.5–5 mg/kg; Gatch and Forster, 2016) or JWH-073 (3–30 mg/kg; Marshell et al., 2014) elicited a dose-dependent reduced locomotor activity. These discrepancies could be due to the species (rat *vs*. mice) difference in response to the treatment or to differences in experimental procedures (e.g., locomotor activity measured 1 h after sc. administration *vs*. measurement immediately after the intraperitoneal treatment). We also found that sc. treatment with Δ^9^-THC (1 or 3 mg/kg) did not affect the total trajectory in the open field test, in line with our previous results (Hlozek et al., 2017).

However, spatial characterization of locomotor behavior showed that Δ^9^-THC (3 mg/kg) increased the time spent in the center of the open field arena. Since this dose did not increase total locomotion, stimulatory effects do not likely account for this; increased exploration of the aversive central zone may therefore suggest an anxiolytic-like effect of this dose. Although there is contradictory literature about the behavioral effects of CB1 receptor activation in animal models of anxiety (as well as in humans), a general conclusion is that low and high doses of CB1 agonists induce anxiolytic and anxiogenic effects, respectively (Moreira and Wotjak, 2010). More specifically, in the elevated plus maze and in the light-dark box test, low doses of Δ^9^-THC in rodents increased the time spent in open arms and the time in the light compartment, respectively (as index of anxiolytic-like effect), through a CB1 mediated mechanism (Berrendero and Maldonado, 2002; Patel and Hillard, 2006; Rubino et al., 2008). By contrast, higher doses of Δ^9^-THC elicited anxiogenic-like responses in rodents (Patel and Hillard, 2006; Rubino et al., 2008; Hlozek et al., 2017). The recent development of cell type specific genetic deletion of CB1 receptors has provided a new tool to better understand cannabinoid action, and assess the different role of the neuronal subpopulations of CB1-expressing neurons, such as GABAergic, glutamatergic and dopamine D1 terminals in the control of emotional behavior (Terzian et al., 2011, 2014; Micale et al., 2017). Given that CB1 receptors on GABAergic *vs*. glutamatergic terminals are required for the anxiogenic- *vs*. anxiolytic-like effects induced by high *vs*. low doses of the CB1 agonist CP55,940 (Rey et al., 2012), we cannot exclude that an anxiolytic-like effect of Δ^9^-THC could be due to the specifically target the CB1 receptors on glutamatergic terminals. Further studies on animals with specific deletion of CB1 receptors in specific neuronal subpopulations are required to support this hypothesis.

In our study, we did not find significant alteration of ASR or PPI induced by acute treatment with Δ^9^-THC, JWH-073 or JWH-210. It is noteworthy that previous studies describe controversial results in relation to the acute effects of cannabinoids on PPI. More specifically, in some studies Δ^9^-THC and SCs (i.e., JWH-073, JWH-18, JWH-250, or WIN55212,2) dose-dependently decreased ASR (Levin et al., 2014; Ossato et al., 2016; Hlozek et al., 2017); as well as in other studies Δ^9^-THC did not affect PPI (Malone and Taylor, 2006; Boucher et al., 2007; Long et al., 2010). Beyond the different route of administration or the different species (rats *vs*. mice) or stress sensitivities, we cannot also exclude that PPI alteration induced by cannabinoid exposure could be strain related, since spontaneously hypertensive rats (SHR), but not Wistar rats had a disturbed PPI induced by CB1/CB2 agonist (Levin et al., 2014).

## Conclusions

Although JWH-073 and JWH-210 at the dose of 0.5 mg/kg had lowest and highest serum levels 1 h after the administration, respectively; our results suggest that their levels are not strictly related to their effects on locomotor activity in our experimental condition. Further evaluation of locomotor activity under different conditions (i.e., higher light intensity an index of aversive condition) is needed. By contrast, Δ^9^-THC at the dose of 3 mg/kg induced anxiolytic-like effect, which seems to be related to its higher serum concentration. Overall, we cannot also exclude that the lack of more significant behavioral effects induced by SCs could be due to their lower serum concentration as compared to Δ^9^-THC. Further behavioral tests are necessary to support the potential therapeutic of endocannabinoid system modulation in the treatment of anxiety disorders (Micale et al., 2013a).

## Author contributions

All authors made a substantial contribution to the conception or design of the work; or the acquisition, analysis, or interpretation of data for the work. All authors were involved in drafting the work or revising it critically for important intellectual contents. All authors gave final approval for the current version of the work to be published. All authors agree to be accountable for all aspects of the work in ensuring that questions related to the accuracy or integrity of any part of the work are appropriately investigated and resolved.

### Conflict of interest statement

The authors declare that the research was conducted in the absence of any commercial or financial relationships that could be construed as a potential conflict of interest. The reviewer MADP and handling Editor declared their shared affiliation.
